# Insights into the Role of Proteolytic and Adhesive Domains of Snake Venom Metalloproteinases from *Bothrops* spp. in the Control of *Toxoplasma gondii* Infection

**DOI:** 10.3390/toxins17020095

**Published:** 2025-02-18

**Authors:** Samuel C. Teixeira, Thales A. M. Fernandes, Guilherme de Souza, Luana C. Luz, Marina Paschoalino, Joed P. de L. Junior, Alessandra M. Rosini, Aryani F. F. Martínez, Vitor de Freitas, Daiana S. Lopes, Patrícia B. Clissa, Vinícius C. de Souza, Milton Y. Nishiyama-Jr., Bellisa F. Barbosa, Eloisa A. V. Ferro, Veridiana de M. R. Ávila

**Affiliations:** 1 Laboratory of Immunophysiology of Reproduction, Institute of Biomedical Sciences, Universidade Federal de Uberlândia, Uberlândia 38405-318, MG, Brazil; samuel.teixeira@ufu.br (S.C.T.); guisbio@hotmail.com (G.d.S.); luanacarvalholuz.28@gmail.com (L.C.L.); marinapaschoalino@gmail.com (M.P.); joedjunior07@gmail.com (J.P.d.L.J.); monteirorosini_alessandra@hotmail.com (A.M.R.); felixafajardo08@gmail.com (A.F.F.M.); bellisafb@ufu.br (B.F.B.); 2 Laboratory of Applied Toxinology, Butantan Institute, São Paulo 05503-900, SP, Brazil; t.fernandes.proppg@proppg.butantan.gov.br (T.A.M.F.); vinicius.souza.esib@esib.butantan.gov.br (V.C.d.S.); milton.nishiyama@butantan.gov.br (M.Y.N.-J.); 3 Laboratory of Biochemistry and Animal Toxins, Institute of Biotechnology, Universidade Federal de Uberlândia, Uberlândia 38405-318, MG, Brazil; vitor_freitas_@live.com; 4 Institute Multidisciplinary in Health, Universidade Federal da Bahia (UFBA), Vitória da Conquista 45029-094, BA, Brazil; lsdaiana@ufba.br; 5 Laboratory of Immunopathology, Butantan Institute, São Paulo 05503-900, SP, Brazil; patricia.clissa@butantan.gov.br

**Keywords:** toxoplasmosis, *Toxoplasma gondii*, snake venom metalloproteinases

## Abstract

Toxoplasmosis is an alarming public health problem that affects more than one-third of the world’s population. In our work, we investigated the antiparasitic effects of catalytically active [BpMP-I and Jararhagin (Jar)] and catalytically inactive [Jararhagin-C (Jar-C)] snake venom metalloproteinases (SVMPs) in human HeLa cells. These toxins impaired the parasite invasion and intracellular growth, and modulated IL-6, IL-8, and MIF cytokines that control the cell susceptibility and response against *T. gondii*. Furthermore, we verified that the antiprotozoal activities are not restricted to the presence of the proteolytic domain, and the adhesive domains participate in the control of *T. gondii* infection. Also, by analyzing the structures of Jar and Jar-C through molecular modeling and dynamics, we observed that the adhesive domains in Jar-C are more exposed due to the absence of the proteolytic domain, which could favor the interaction with different targets. Our investigation on the role of SVMP domains in combating *T. gondii* infection highlights their potential application as biotechnological tools for creating more effective treatments for toxoplasmosis.

## 1. Introduction

*Toxoplasma gondii* is an obligate intracellular parasite capable of infecting a wide variety of nucleated cells and tissues [1,2]. It is estimated that 30 to 50% of the global population is affected by *T. gondii*; the prevalence of this protozoosis may vary according to the region, and these differences are associated with diet, water treatment, intensity of environmental exposure, cultural habits, and socioeconomic factors [3,4].

*T. gondii* infection can lead to severe symptoms in immunocompromised individuals and children [5]. Congenital transmission can be systemic, leading to stillbirth, fetal death, miscarriage, intracranial calcifications, neurological impairments, and ocular conditions [6,7]. Congenital toxoplasmosis is a significant public health issue worldwide, including in Brazil, where it is classified as a neglected disease [8].

The gold standard treatment for toxoplasmosis involves the combination of sulfadiazine and pyrimethamine; however, adverse effects associated with these drugs include hematological alterations, such as anemia, thrombocytopenia, leukopenia, hypersensitivity reactions, and an increased risk of kidney stones and hepatic complications due to potential bone marrow suppression [9,10,11,12]. For pregnant women, the use of pyrimethamine has been linked to teratogenic effects and is recommended only after the first trimester of pregnancy [13,14]. In addition, numerous studies have demonstrated that *T. gondii* possesses remarkable adaptive potential, leading to the emergence of drug-resistant parasites [15,16,17]. Therefore, the search for more effective and less toxic drugs is necessary for the prevention and treatment of toxoplasmosis.

In this context, a variety of compounds isolated from natural sources have been used for drug development, due to their prominent biological properties [18]. Among them, snake venoms have emerged for the development of broad-spectrum therapeutic molecules, as they are composed of a wide variety of biologically active compounds, such as enzymatic and non-enzymatic molecules [19,20]. Despite the well-known lethal effects, snake venom toxins exert a variety of prominent effects, such as antimicrobial [21], antitumor [22], antiviral [23,24], and antiparasitic [25,26] activities. These characteristics have increased the interest of many research groups in deeply exploring the structural–functional relationship of these bioactive molecules, focusing on its medicinal potential [18].

Among the most common and well-studied toxins, snake venom metalloproteinases (SVMPs) have been highlighted. These enzymes are members of the M12B clan of metalloproteinases, which includes the family ADAM (A Disintegrin And Metalloproteinase), and are categorized into three classes (P-I, P-II, and P-III) [27]. P-I presents a single catalytic metalloproteinase (M) domain dependent on Zn^2+^; P-II comprises an M domain and a disintegrin-like (D) domain; and P-III possesses the M domain, a D domain and a cysteine-rich (C) domain [27].

BpMP-I is a P-I SVMP of 20 kDa isolated from *B. pauloensis* snake venom. This toxin is a fibrinogenolytic and anticoagulant metalloproteinase with non-hemorrhagic activities [28]. Jar is a P-III SVMP composed of 421 amino acids obtained from *B. jararaca* snake venom, with a predicted molecular mass of 47 kDa and an estimated 52 kDa in polyacrylamide gel electrophoresis due to the glycosylation at Asn183 [29]. Jar-C is a protein of 28 kDa originated from the proteolytic processing of Jar consisting of the D and C domains without known glycosylation sites [30,31].

Bastos and colleagues [32] pioneeringly reported the anti-*T. gondii* effects of neuwiedase, a P-I metalloproteinase isolated from *Bothrops neuwiedi*, on fibroblast cells, as a model of systemic toxoplasmosis. The authors demonstrated that safe concentrations of neuwiedase impaired both *T. gondii* invasion and replication, as well as modulated the immune response [32]. In order to gain insights into the underlying antiparasitic activities of SVMPs, BpMP-I (P-I), Jararhagin (P-III), and Jararhagin-C (a disintegrin-like protein) were used to investigate the involvement of the proteolytic and adhesive domains in controlling *T. gondii* infection.

## 2. Results

### 2.1. SVMPs Did Not Alter Cell Viability Even at Higher Concentrations

**Figure 1:**
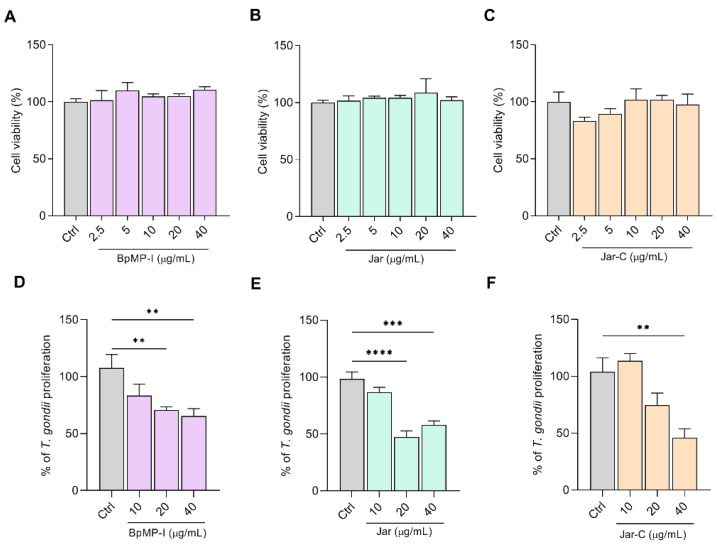
Cell viability and *T. gondii* proliferation assays treated with snake venom metalloproteinases. Cell viability (MTT) (**A**–**C**) and *T. gondii* proliferation (**D**–**F**) with different concentrations of the metalloproteinases: BpMP-I (**A**,**D**), Jar (**B**,**E**), and Jar-C (**C**,**F**) (ranging from 40 to 2.5 µg/mL for the viability assay; or from 40 to 10 µg/mL for the parasite proliferation assay). For both experiments, RPMI 1640 medium served as the negative control (Ctrl). Data were obtained from three independent experiments, each consisting of 8 replicates. The results are expressed as the mean percentage (%) of cell viability for the MTT assay ± standard error of the mean (SEM); and the mean percentage (%) of proliferation ± standard error of the mean (SEM) of *T. gondii* for the proliferation assay. Statistical analyses were performed using one-way ANOVA and Dunn’s multiple comparison test to identify significant differences between groups (*p* < 0.05). The negative control of both assays was considered 100% cell viability or 100% *T. gondii* proliferation. The significance levels are denoted as **** for *p* < 0.0001, *** for *p* < 0.001, and ** for *p* < 0.01.

Firstly, we evaluated the impact of SVMPs on the viability of HeLa cells, treating them with increasing concentrations of BpMP-I, Jar, and Jar-C for 24 h. Any concentration tested (2.5 to 40 µg/mL) did not cause a loss of viability compared to the control group (Ctrl, untreated group) (Figure 1A–C).

### 2.2. BpMP-I, Jar, and Jar-C Significantly Decreased the Intracellular Proliferation of T. gondii

To determine the possible effective concentrations against *T. gondii*, we selected the three highest concentrations of each SVMPs (10, 20, and 40 µg/mL) for further antiparasitic assays.

After the incubation of *T. gondii*-infected cells with SVMPs, our data revealed that BpMP-I (20 and 40 µg/mL; ** *p* < 0.01) and Jar (20 and 40 µg/mL; **** *p* < 0.0001 and *** *p* < 0.001, respectively) were capable of inhibiting parasite growth in host cells in comparison with the control group (Ctrl, Figure 1D,E). Regarding the Jar-C treatment, parasite proliferation was significantly controlled with 40 µg/mL (** *p* < 0.01), when compared to the control group (Ctrl, Figure 1F).

### 2.3. SVMP Treatments Differently Modulated the Concentrations of Cytokines

**Figure 2:**
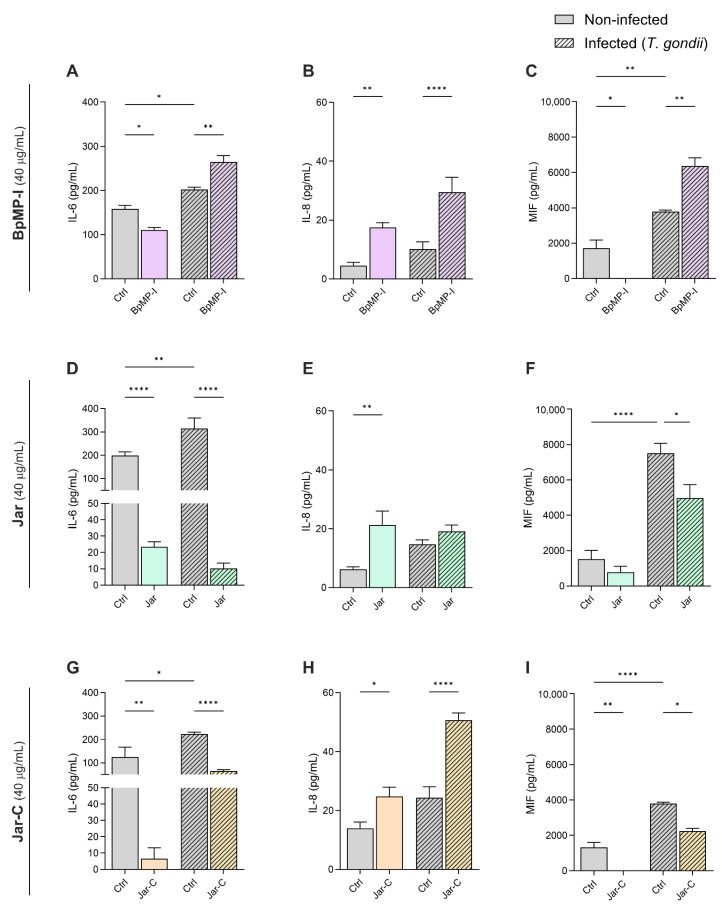
Evaluation of cytokines in HeLa cells infected with *T. gondii* after a 24 h treatment with SVMPs. HeLa cells were infected or not with *T. gondii* tachyzoites and subsequently treated with the metalloproteinases (40 µg/mL): BpMP-I (**A**–**C**), Jar (**D**–**F**), and Jar-C (**G**–**I**). After that, the levels of the cytokines IL-6 (**A**,**D**,**G**), IL-8 (**B**,**E**,**H**), and MIF (**C**,**F**,**I**) were quantified by the ELISA method. As the negative control (Ctrl), RPMI 1640 medium was used. Data were obtained from three independent experiments, each one comprising 8 replicates, and the results are expressed as mean ± standard error of the mean (SEM). Statistical analyses were performed using one-way ANOVA and Šídák’s multiple comparison test to identify significant differences between groups (*p* < 0.05). The significance levels are denoted as **** for *p* < 0.0001, ** for *p* < 0.01, and * for *p* < 0.05.

In the BpMP-I treatment, we observed a reduction in both IL-6 and MIF levels in uninfected HeLa cells compared to the uninfected/untreated cells (* *p* < 0.05); conversely, BpMP-I treatment increased both cytokines production by infected cells in relation to the infected/untreated cells (** *p* < 0.01) (Figure 2A–C). Figure 2B demonstrates that BpMP-I upregulated the IL-8 release by HeLa cells in comparison to uninfected/untreated (* *p* < 0.05) or infected/untreated (*** *p* < 0.001) cells.

Regarding Jar treatment, this SVMP strongly downmodulated IL-6 release by HeLa cells in the presence or absence of infection when compared to the uninfected/untreated or infected/untreated cells (*** *p* < 0.001) (Figure 2D). In addition, Jar treatment promoted an augmentation of IL-8 levels in comparison to the uninfected/untreated group (** *p* < 0.01) (Figure 2E). Also, Jar reduced MIF release by HeLa cells in relation to the infected/untreated group (* *p* < 0.05) (Figure 2F).

Concerning the Jar-C, the toxin decreased both IL-6 and MIF levels in uninfected HeLa cells compared to the uninfected/untreated cells (** *p* < 0.01); similarly, Jar-C downmodulated both IL-6 (**** *p* < 0.0001) and MIF (* *p* < 0.05) production by *T. gondii*-infected cells in relation to the infected/untreated cells (Figure 2G–I). In contrast, Jar-C upregulated IL-8 production by HeLa cells in comparison to uninfected/untreated (* *p* < 0.05) or infected/untreated (**** *p* < 0.0001) cells (Figure 2H).

In *T. gondii* infection, our findings reported that the parasitic infection caused an augmentation of IL-6 (Figure 2A,D,G) and MIF levels (Figure 2C,F,I).

TNF cytokine was not detected in supernatants under any experimental conditions.

### 2.4. Jar and Jar-C Directly Targeted the Host Cell and T. gondii Tachyzoites, Impairing the Establishment of Infection

**Figure 3:**
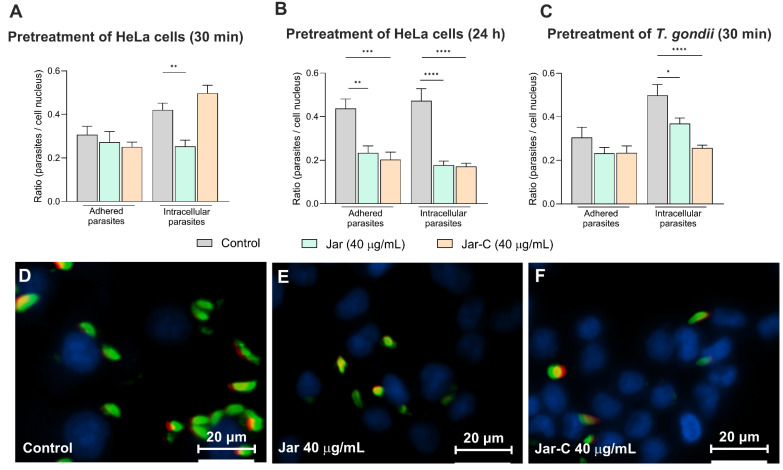
Assessment of snake venom metalloproteinase activity on *T. gondii* adhesion and invasion in HeLa cells. HeLa cells were subjected to pretreatment with Jar-C or Jar (40 μg/mL) for 30 min (**A**) or 24 h (**B**) and then infected with *T. gondii* tachyzoites for 3 h. Alternatively, *T. gondii* tachyzoites were previously treated for 30 min with Jar-C or Jar (40 μg/mL) before being allowed to infect HeLa cells for 3 h (**C**). The images (**D**–**F**) were captured using a confocal microscope and display the adhered parasites stained with Alexa Fluor 488 conjugated anti-mouse IgG (green). HeLa cells nuclei were counterstained using DAPI (blue). Intracellular and adhered parasites are shown in green and red/yellow, respectively. As the negative control (Ctrl), RPMI 1640 medium supplemented with 5% FBS was employed. The dataset was derived from three independent experiments, each comprising 8 replicates, and the results are expressed as mean ± standard error of the mean (SEM). Statistical analyses were performed using one-way ANOVA and Šídák’s multiple comparison test to identify significant differences between groups (*p* < 0.05). The significance levels are indicated as **** for *p* < 0.0001, *** for *p* < 0.001, ** for *p* < 0.01, and * for *p* < 0.05.

Since BpMP-I and Jar (both catalytically active) and Jar-C (catalytically inactive) demonstrated prominent anti-*T. gondii* action, we assessed the ability of Jar or Jar-C to interfere with the early steps of the lytic cycle of the parasite (i.e., adhesion and invasion processes) to gain insights into the involvement of the SVMP domains in the control of *T. gondii* infection. Thus, we promoted both the pretreating of the cells and the parasites before infection. In the first set of experiments, HeLa cells were pretreated with Jar or Jar-C (40 μg/mL) for 30 min or 24 h and then infected with *T. gondii* for 3 h. As observed, the pretreatment of HeLa cells with Jar for 30 min caused a significant reduction in the number of intracellular parasites (** *p* < 0.01) (Figure 3A). Similarly, the pretreatment for 24 h with Jar (** *p* < 0.01) and Jar-C (*** *p* < 0.001) promoted a decrease in the adhered parasites compared to the control group; similarly, both SVMPs diminished the number of intracellular parasites (**** *p* < 0.0001) in comparison to the control group, suggesting a protective effect on HeLa cells (Figure 3B).

In the second set of experiments, tachyzoites were previously treated with Jar or Jar-C (40 μg/mL) for 30 min before being allowed to interact with HeLa cells. Our results demonstrated that Jar (* *p* < 0.05) and Jar-C (**** *p* < 0.0001) significantly reduced the number of intracellular parasites compared to the untreated parasites (Figure 3C). Representative images highlighting the impact of Jar and Jar-C treatments for 24 h on HeLa cells in the tachyzoites–host cell interaction are shown in Figure 3D–F.

### 2.5. EDTA-Mediated Inhibition of the Proteolytic Activity of BpMP-I and Jar Completely Abolished Their Abilities to Control T. gondii Growth

**Figure 4:**
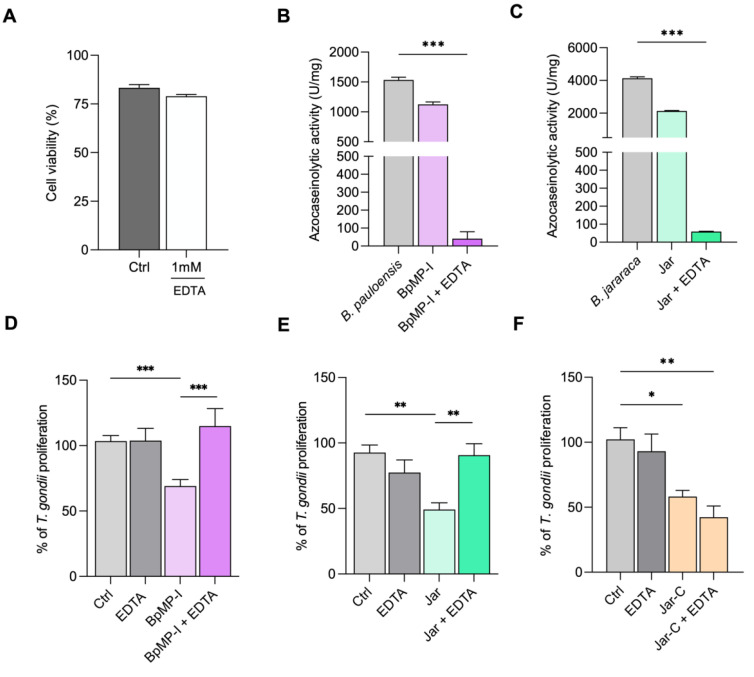
Evaluation of azocaseinolytic activity and analysis of enzymatic domain dependence in controlling *T. gondii* infection. HeLa cell viability following a 24 h treatment with 1 mM of EDTA (**A**). Azocaseinolytic activity of BpMP-I (**B**) and Jar (**C**) was evaluated in the presence or absence of EDTA. For this assay, BpMP-I and jararhagin metalloproteinases were incubated with azocasein, both with and without EDTA. Results are presented as a unit (U) of azocaseinolytic activity, defined as an increase of 0.01 absorbance units per minute. The proliferation of *T. gondii* in HeLa cells treated with BpMP-I (**D**), Jar (**E**), and Jar-C (**F**) in conjunction with EDTA was also examined. In this assay, HeLa cells were initially infected with *T. gondii* tachyzoites for 3 h and then treated for 24 h with the respective metalloproteinases + EDTA. RPMI 1640 medium supplemented with 5% FBS and EDTA alone were employed as controls. Data were obtained from three independent experiments, each comprising 8 replicates, and the results are expressed as mean ± standard error of the mean (SEM). Statistical analyses were performed using the unpaired *t*-test (**A**), one-way ANOVA, and Dunn’s (**B**,**C**) or Šídák’s (**D**–**F**) multiple comparison tests to identify significant differences between groups (*p* < 0.05). The significance levels are presented as *** for *p* < 0.001, ** for *p* < 0.01, and * for *p* < 0.05.

Firstly, we demonstrated that the concentration of EDTA (1 mM) used for proteolytic inhibition did not show toxicity against HeLa cells (Figure 4A). As expected, the previous treatment of the catalytically active SVMPs (BpMP-I and Jar) with EDTA (1 mM) caused a strong enzymatic inhibition upon azocasein substrate compared to the crude venom of *B. pauloensis* and *B. jararaca*, respectively, which means that their catalytic sites were successfully inactivated (*** *p* < 0.001) (Figure 4B,C).

Next, we assessed whether BpMP-I, Jar, or Jar-C would be able to maintain their ability to control parasite intracellular proliferation even in the presence of EDTA. As previously demonstrated, BpMP-I (*** *p* < 0.001), Jar (** *p* < 0.01), and Jar-C (* *p* < 0.05) controlled parasite growth compared to the infected/untreated cells (Figure 4D–F). EDTA completely impaired the capacities of BpMP-I (*** *p* < 0.001) and Jar (** *p* < 0.01) in restricting the parasite replication in comparison to the respective toxin-treated group (Figure 4D,E). As expected, Jar-C maintained its anti-*T. gondii* effect even in the presence of EDTA in relation to the control group (untreated cells) (** *p* < 0.01) (Figure 4F), since this toxin is devoid of enzymatic activity.

### 2.6. In Silico Analyses Shed Light into the Structure of the Jar and Jar-C

The primary structure of Jar shares 95.47% of identity with Bothropasin, presenting mutations mainly located in the N-terminal of the M domain. In parallel, Jar-C and Bothropasin share 99.53% of identity, differing by one single amino acid in the N-terminus of Jar-C. Jar is organized in accordance with its template Bothropasin in the M domain (Glu^1^-Asp^209^), D domain (Ile^210^-Lys^302^), and C domain (Asn^303^-Tyr^421^), while Jar-C is formed by the identical D and C domains of Jar and begins at Ile^210^. The MSA can be found in the Supplementary File S3.

**Figure 5:**
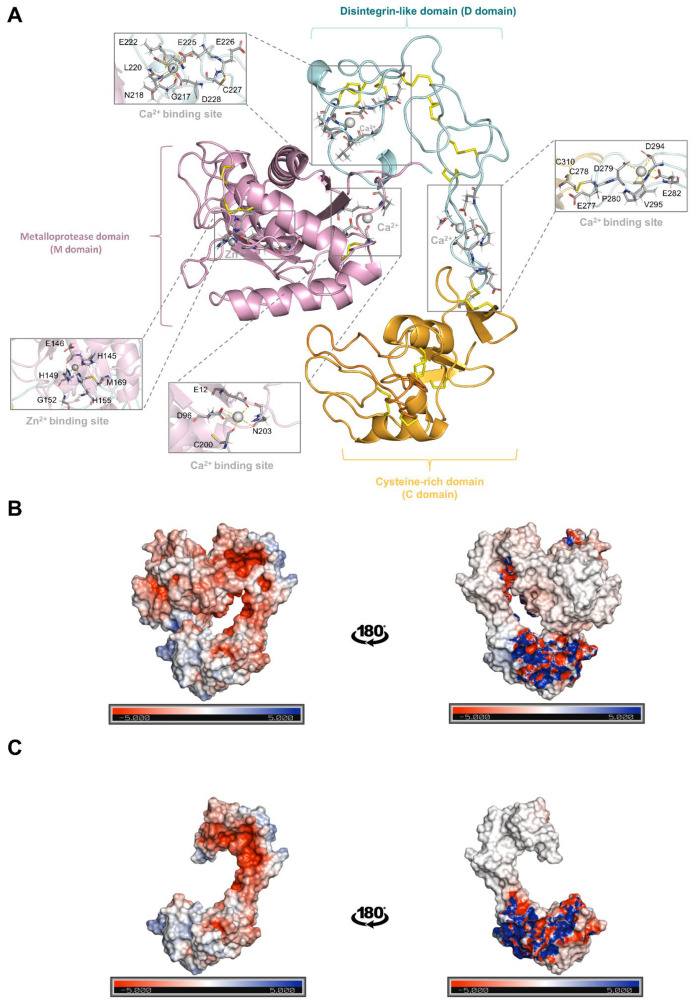
Molecular modeling of Jar and Jar-C. (**A**) Jar and Jar-C domains. The metalloproteinase (M), disintegrin-like (D) and cysteine-rich (C) domains are represented in the image. The M domain is colored in pink, the D domain in light blue and the C domain in orange. Jar is formed by the M, D and C domains, while Jar-C is formed by the identical D and C domains. Disulfide bonds are shown as yellow sticks. The Zn^2+^ and Ca^2+^ binding sites are evidenced by gray boxes. The Zn^2+^ and Ca^2+^ are represented as spheres and the residues in the binding site as sticks, with the carbon, hydrogen, nitrogen, and oxygen atoms in gray, white, blue, and red, respectively. Electrostatic surface of Jar (**B**) and Jar-C (**C**). The blue, white, and red colors indicate positive, neutral, and negative charges, respectively.

The molecular modeling of Jar showed that the D and the C domain form a concave surface toward the M domain (Figure 5A). The M domain of Jar presents a conserved Ca^2+^ binding site and three disulfide bonds (Cys^120^-Cys^200^, Cys^160^-Cys^184^, and Cys^162^-Cys^167^) (Figure 5A). The D domain of Jar and Jar-C contains seven disulfide bonds (Cys^216^-Cys^245^, Cys^227^-Cys^240^, Cys^229^-Cys^235^, Cys^239^-Cys^262^, Cys^253^-Cys^259^, Cys^258^-Cys^284^, and Cys^271^-Cys^291^) and two conserved Ca^2+^ binding sites (Figure 5A). In addition, this domain accommodates the disintegrin-like loop (Glu^226^-Cys^227^-Asp^228^) (Figure 5A). The C domain of Jar and Jar-C present six disulfide bonds (Cys^303^-Cys^315^, Cys^322^-Cys^372^, Cys^337^-Cys^383^, Cys^350^-Cys^360^, Cys^367^-Cys^409^, and Cys^403^-Cys^414^) (Figure 5A). Despite the lack of the M domain, the D and C domains of Jar-C conserved the C-shaped format, which is reflected by the low RMSD value of 0.022 Å. Interestingly, the protein surface inspection showed that in Jar-C, the D and C domains are more exposed given the absence of the M domain (Figure 5B,C).

**Figure 6:**
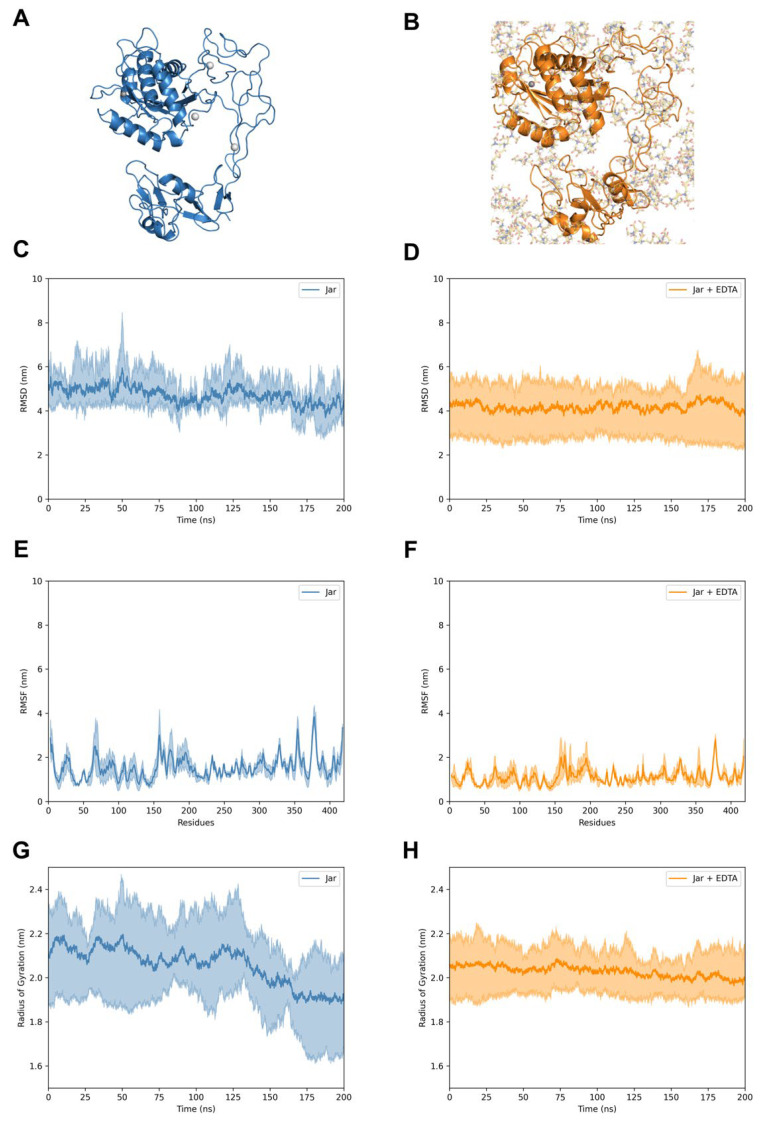
Molecular dynamics of Jar. Structures of Jar in the absence (**A**) and presence (**B**) of EDTA. The proteins and EDTA are represented in the image and sticks, respectively. For clarity, the water molecules and Na^+^ and Cl^−^ ions were hidden. RMSD (nm) of Jar in the absence (**C**) and presence (**D**) of EDTA. RMSF (nm) of Jar without (**E**) and with (**F**) EDTA. Radius of gyration (nm) of Jar in the absence (**G**) and presence (**H**) of EDTA. The central lines indicate the average values among the simulations contoured by the maximum and minimum values.

**Figure 7:**
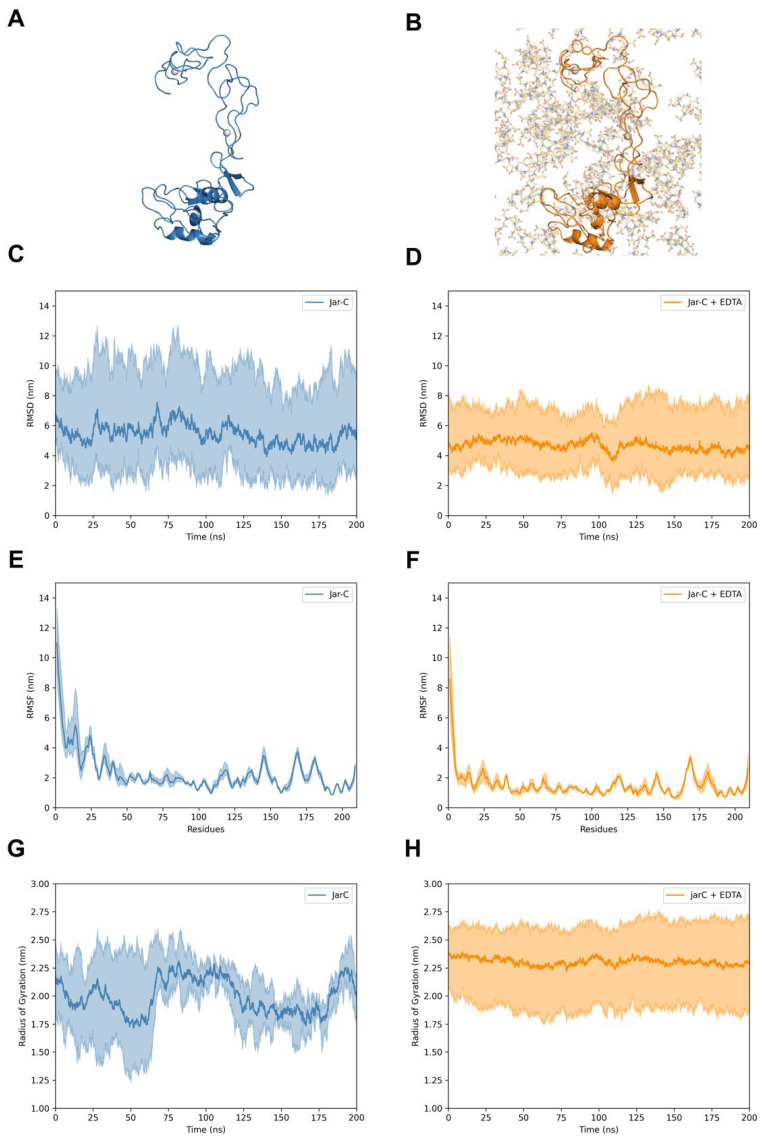
Molecular dynamics of Jar-C. Structures of Jar-C in the absence (**A**) and presence (**B**) of EDTA. The proteins and EDTA are represented in the image and sticks, respectively. For clarity, the water molecules and Na^+^ and Cl^−^ ions were hidden. RMSD (nm) of Jar-C in the presence (**C**) and absence (**D**) of EDTA. RMSF (nm) of Jar-C without (**E**) and with (**F**) EDTA. Radius of gyration (nm) of Jar-C in the presence (**G**) and absence (**H**) of EDTA. The central lines indicate the average values among the simulations contoured by the maximum and minimum values.

Through molecular dynamics, we explored the behavior of Jar and Jar-C and the impact of EDTA on their structures. Although the molecular modeling showed a remarkable similarity between Jar and Jar-C, we noticed that Jar (Figure 6A,B) presents a less dynamic structure than Jar-C (Figure 7A,B). Moreover, despite the fact that Jar-C contains the adhesive domains of Jar, we observed that the D domain was less flexible in Jar (Figure 6C) than in Jar-C (Figure 7C). In fact, the D domain established a scaffold connecting the M and C domains of Jar. The presence of the Ca^2+^ ions and the disulfide bonds may contribute to stabilizing the structure of the D and C domains. In parallel, we verified that the simulations with EDTA caused the loss of the Zn^2+^ but not the Ca^2+^ ions and introduced small variations in the structure of Jar (Figure 6D–F) and Jar-C (Figure 7D–F). The recordings of molecular dynamics simulations can be found in the Supplementary Materials.

## 3. Discussion

Toxoplasmosis is an alarming public health problem with harmful consequences that affects more than one-third of the world’s population [33]. The standard treatment for toxoplasmosis consists of the use of sulfadiazine and pyrimethamine; however, this therapy is often accompanied by adverse side effects [9,10,11,12,13,14]. Therefore, the search for more effective molecules is necessary for the prevention and control of toxoplasmosis.

Snake venoms have emerged as a valuable source of molecules with pharmacological potential [34], including antiprotozoal activities [35]. In this context, this study investigated the effects of three SVMPs (BpMP-I, Jar, and Jar-C) isolated from *Bothrops* spp. snake venoms against *T. gondii* infection in HeLa cells, as well as explored the role of the SVMPs domains in their antiparasitic action.

Although metalloproteinases represent an important component of viperid venoms, limited attention has been given to their antiparasitic properties [35]. Nevertheless, a handful of studies have demonstrated the prominent activities against protozoan parasites, such as *P. falciparum* and *T. gondii* [32,36,37,38].

To explore the actions of SVMPs against *T. gondii*, we sought to investigate the role of SVMP domains in impairing the early steps of the *T. gondii* lytic cycle. Our findings revealed that the pretreatment of both cells or parasites before infection with Jar and Jar-C compromised the parasite invasion, suggesting that both SVMPs exert their antiparasitic activities by targeting parasites and/or cells in the early stages of infection. Bastos et al. [32] demonstrated that neuwiedase (P-I SVMP) inhibited *T. gondii* invasion and replication on previously infected host cells or on parasites before fibroblast infection. In our study, we showed that BpMP-I, Jar, and Jar-C restricted *T. gondii* growth in HeLa cells. Noteworthy, the antiparasitic effects of these SVMPs were not exclusively dependent on their proteolytic activity, since Jar-C (catalytically inactive) was also able to impair *T. gondii* intracellular proliferation, suggesting that the adhesive domains participate in the antiparasitic activities.

In order to assess the possible involvement of the host immune response triggered by the SVMP treatments impairing *T. gondii* infection, we measured the cytokine levels released by HeLa cells. In general, Jar treatment downregulated the release of IL-6 and MIF by infected cells, while BpMP-I upregulated the levels of IL-6, IL-8, and MIF, and Jar-C increased IL-8 production in *T. gondii*-infected cells, which contributed to the reduction in parasite intracellular growth given the role of these cytokines in the control of *T. gondii* proliferation [39,40,41,42].

Clissa et al. (2001) [43] reported that Jar positively regulates the IL-1β, IL-6, and TNF-α expression at the transcriptional levels, but not at the protein level. Furthermore, the authors demonstrated that Jar degraded soluble cytokines by proteolytic activity. In parallel, Silva et al. (2016) [44] showed that BooMP-Alpha-I, a P-I SVMP from *Bothrops moojeni*, promoted the direct proteolysis of TNF, indicating that zinc metalloproteinases can modulate TNF levels. Hence, the negative regulation of IL-6 mediated by Jar treatment in HeLa cells could be a consequence of the proteolytic action; nevertheless, to validate this hypothesis, further studies need to be thoroughly evaluated.

Taken together, BpMP-I, Jar, and Jar-C differently modulated the levels of IL-8, MIF, and IL-6, which are cytokines associated with the host cell susceptibility to *T. gondii* infection [45,46,47,48,49]. It is worth mentioning that although Jar-C possesses the identical disintegrin-like and cysteine-rich domains of Jar, the cytokine levels were differently modulated.

Jar belongs to the P-III class of SVMPs and presents multiple effects associated with the D and C domains [50,51,52]. The M domain of P-III SVMPs is involved in the proteolysis of extracellular components, the processing of cytokines, and the shedding of integrins from cell surfaces [50,51,52]. Due to differences in effects among P-III SVMPs, it is proposed that the D and C domains also play important roles in biological effects [50,51,52]. Despite the absence of the M domain, Jar-C possesses relevant activities, including the inhibition of collagen-induced platelet aggregation [30], proinflammatory effects [53], leukocyte recruitment [54,55], and binding to the basement membrane collagens within the vascular lesion [56].

To gain insights into the role of the proteolytic domain in controlling *T. gondii* infection, we preincubated BpMP-I, Jar, and Jar-C with EDTA, a chelator of divalent cation. The EDTA-mediated inhibition of the catalytic activity of BpMP-I and Jar completely abolished their ability to regulate the parasite intracellular proliferation. Therefore, the anti-*T. gondii* effects of both BpMP-I and Jar are probably dependent on the catalytic mechanism. In parallel, Jar-C continued impairing the parasite growth even in the presence of EDTA, pointing out that the D and C domains of this SVMP are involved in their antiprotozoal effect.

It has been previously shown that the inhibition of the catalytic activity of Jar is not sufficient to abolish its effects, highlighting that the disintegrin-like/cysteine-rich domains are able to interact with host cell components, triggering biological effects [51]. Since the D and C domains of Jar-C are also present in Jar, we raised the following question: why is it that it did not maintain its antiparasitic effect in the presence of EDTA? In this intriguing scenario, we further employed in silico analyses with Jar and Jar-C (with or without EDTA) to shed light on the differences in the anti-*T. gondii* activities.

The tertiary structure of Jar showed that the protein conserves the C-shaped format of P-III SVMPs, similarly to Bothropasin [30]. In parallel, despite the lack of the M domain, Jar-C maintained the D and C domains in the C-shaped conformation, which may be stabilized by the conserved disulfide bonds and Ca^2+^ ions [30]. In addition, through molecular modeling and dynamics, we observed that, due to the absence of the catalytic domain, the D and C domains of Jar-C are more exposed. These adhesive domains present conserved protein–protein interaction sites among different SVMPs, which mediates the binding of molecular targets involved in their biological activities, including fibronectin, vWF (von Willebrand factor), and integrins [57,58,59,60,61,62]. Interestingly, *T. gondii*’s ability to invade host cells involves an attachment to integrin receptors [63,64,65], which could be a target involved in the anti-*T. gondii* effects mediated by SVMPs. In view of the differences in the structures and effects of Jar and Jar-C, we suggest that the accessibility of the domains may impact the binding to distinct targets, triggering different mechanisms of action in the control of the *T. gondii* infection. Moreover, as glycosylation is a major protein modification of snake venom toxins and plays important roles in their structures and activities [66,67,68,69,70,71,72,73,74,75], this important post-translational modification (PTM) could influence the activity of the toxins studied, as Jar is known to be glycosylated at the proteolytic domain, and Jar-C lacks predicted glycosylation sites [30,31].

The use of metal chelators contributes to the understanding of the behavior of the SVMP domains and to the development of more effective therapeutic agents [76]. EDTA not only inhibits the enzymatic activity of SVMPs but can alter the structure by producing aggregates and introducing structural alterations [77,78,79,80]. Interestingly, Zychar et al. [54] verified that 1,10-phenanthroline (oPhe), a zinc chelator, caused enzymatic inactivation and structural modifications of Jar without preventing the binding to collagen, pointing out that the M domain is essential for Jar activity, as the inactivated enzyme did not promote leukocyte–endothelial cell interactions [54]. Also, the authors verified that, despite the absence of the M domain, Jar-C had an effect on the cellular interactions, indicating that the D or the C domain could also participate in inflammatory responses [54]. In view of this, the absence of the antiparasitic activity of Jar in the presence of EDTA could be partially explained due to the enzymatic inactivation of the M domain, while Jar-C maintained the anti-*T. gondii* effect through the D and C domains.

Taken together, Jar and Jar-C may interact with different targets in *T. gondii*, triggering distinct mechanisms of action. In Jar, the D and/or C domains may be involved in the substrate recognition for the proteolytic action of the M domain. In Jar-C, the absence of the catalytic domain makes the adhesive domains more accessible and may facilitate interactions with distinct targets. Given their prominent antiparasitic effects, a better comprehension of the functional role of each domain in the control of *T. gondii* infection would corroborate the use of these toxins as biotechnological tools for the selection of molecular targets, helping the development of alternative approaches for the treatment of toxoplasmosis.

## 4. Conclusions

In summary, we assessed the anti-*T. gondii* effects of three SVMPs (BpMP-I, Jar, and Jar-C) from *Bothrops* spp. These toxins impaired both the *T. gondii* invasion and intracellular proliferation, as well as modulated different key cytokines involved in the control of *T. gondii* infection. Moreover, we demonstrated that the effects are not fully dependent on the proteolytic action, and the adhesive domains are also involved in the antiprotozoal activity. Our results corroborate the potential use of SVMPs as a biotechnological tool for the search of more effective and less toxic molecules against toxoplasmosis.

## 5. Materials and Methods

### 5.1. Venoms and Toxins

*Bothrops* crude venoms were collected from snakes kept at the CETA (Animal Toxin Extraction Center Ltd.a.—CNPJ: 08.972.260/0001-30, Morungaba, SP, Brazil), which has received IBAMA registration and approval for the use of renewable natural resources (n° 2087163). BpMP-I was supplied by the Biochemistry and Animal Toxins laboratory at the Federal University of Uberlândia. The toxin was purified from freeze-dried *B. pauloensis* venom according to Naves de Souza et al. (2012) [28].

Jararhagin (Jar) and Jararhagin-C (Jar-C) were purified from the freeze-dried venom of *Bothrops jararaca*, supplied by the Herpetology Laboratory at the Butantan Institute (São Paulo, SP, Brazil). The purification was carried out as described by Ferreira et al. (2018) [49]. Protein concentration was measured using Bradford assay [81]. The analysis of protein purity was evaluated by SDS-PAGE electrophoresis (Supplementary File S1) under denaturing conditions, with minor modifications [82]. In brief, the 12.5% (*w*/*v*) gel for BpMP-I and the 15% (*w*/*v*) gel for Jar and Jar-C were prepared using the Hoefer SE 260 Mighty Small II Mini-Vertical electrophoresis system (Bridgewater, MA, USA). The samples were dispersed in 0.06 M Tris-HCl (LGC Biotecnologia, SP, Brazil) pH 6.8, 0.01% (*w*/*v*) bromophenol blue (Vetec Quimica Fina Ltda., SP, Brazil), 10% (*v*/*v*) glycerol (Synth, SP, Brazil), and 20% (*v*/*v*) β-mercaptoethanol (Sigma Chemical Co., St. Louis, MO, USA). After the run, the gels were stained with a 0.1% (*w*/*v*) Coomassie Brilliant Blue R-250 (Sigma) solution in a 1:1 (*v*/*v*) water and ethanol mixture and destained with a 7% acetic acid solution (L.S. Chemicals, Mumbai, Maharashtra, India).

### 5.2. Cell Culture and Parasite Maintenance

Human uterine cervical (HeLa) cells were obtained from the American Type Culture Collection (ATCC, Manassas, VA, USA) and cultured in 25 cm^2^ flasks containing RPMI 1640 medium, supplemented with 100 U/mL of penicillin, 100 μg/mL of streptomycin (Sigma), and 10% fetal bovine serum (FBS) (Cultilab, Campinas, SP, Brazil). The cells were maintained in a humidified incubator at 37 °C with 5% CO_2_, as previously described [39].

*T. gondii* tachyzoites (2F1 clone) constitutively expressing the β-galactosidase gene and derived from the highly virulent RH strain were given by Dr. Vern Carruthers, from the School of Medicine at the University of Michigan (USA). The parasites were maintained by serial passages in HeLa cells cultured in medium containing 2% FBS, 100 U/mL of penicillin and 100 mg/mL of streptomycin at the same described conditions [39].

### 5.3. Cellular Viability

The effect of each SVMPs on HeLa cells viability was assessed by the tetrazolium salt colorimetric (MTT) assay [83]. HeLa cells were seeded at 3 × 10^4^ cell/well in 100 μL of RPMI 1640 medium with 10% FBS in 96-well microplates. After adhesion, cells were treated for 24 h at 37 °C with twofold serial dilutions of BpMP-I, Jar, and Jar-C (ranging from 40 to 2.5 μg/mL). Only culture medium was used as a positive control of viability, and these cells were considered to be 100% viable. Next, the supernatants were removed and the cells were incubated with MTT reagent (5 mg/mL, 10 μL) (Sigma) plus 90 μL of supplemented RPMI medium for 3 h followed by the addition of 10% sodium dodecyl sulfate (SDS, Sigma) and 50% N,N-dimethylformamide (Sigma). MTT reduction was measured at 570 nm absorbance, and the cell viability was reported in percentages (cell viability %).

### 5.4. T. gondii Intracellular Proliferation Assay

The β-galactosidase colorimetric assay was used to assess BpMP-I, Jar, and Jar-C effects on *T. gondii* intracellular proliferation in HeLa cells, as previously described [84]. In brief, HeLa cells were plated at 3 × 10^4^ cells/well in 96-well microplates and incubated overnight at 37 °C and 5% CO_2_. After the adhesion, cells were infected with *T. gondii* tachyzoites at a multiplicity of infection (MOI) of 3:1 in supplemented RPMI medium with 2% of FBS for 3 h. Then, cells were rinsed with 1× PBS (Sigma) in order to remove the excess of extracellular parasites. Based on MTT assay, we treated the *T. gondii*-infected HeLa cells for 24 h at 37 °C with 10, 20, and 40 μg/mL of each SVMP as the highest non-cytotoxic doses for host cells. The classic combination of sulfadiazine (SDZ, Sigma) and pyrimethamine (PYR, Sigma) was used as a positive control for parasite control. The concentration of SDZ (200 μg/mL) + PYR (8 μg/mL) used was based on a previously published work [85]. In parallel, culture supernatants were collected and stored at −80 °C for the further measurement of cytokines. *T. gondii* intracellular proliferation was screened based on β-galactosidase activity using the chlorophenol red-β-D-galactopyranoside reagent substrate (CPRG; Roche Diagnostics, Mannheim, Germany). In brief, infected HeLa cells were lysed with 100 μL of ice-cold lysis buffer (100 mM of HEPES, pH 8.0, 1 mM of MgSO_4_, 0.1% Triton X-100, and 5 mM of dithiothreitol) (Sigma), and mixed with 160 μL of assay buffer (100 mM of phosphate buffer, pH 7.3, 102 of mM β-mercaptoethanol, and 9 mM of MgCl_2_) (Sigma) and 40 μL of 3.125 mM of CPRG. The number of tachyzoites was obtained according to a reference curve containing free tachyzoites (ranging from 1 × 10^6^ to 15.6 × 10^3^) and data were expressed as percentage of *T. gondii* proliferation (% of *T. gondii* proliferation) in each treatment condition compared to control (infected/untreated cells), which corresponded to 100% of parasite proliferation [85].

### 5.5. Cytokine Measurement

The release of cytokines in the supernatants of HeLa cells was measured using a double-antibody sandwich enzyme-linked immunosorbent assay (ELISA). Assays for IL-6, IL-8, TNF (OpTEIA, BD Biosciences, San Diego, CA, USA), and MIF (Duoset R&D Systems, Minneapolis, MN, USA) were conducted following the manufacturer’s instructions. Cytokine measurement was made at 450 nm absorbance with a multi-well scanning spectrophotometer (Titertek Multiskan Plus, Flow Laboratories, McLean, VA, USA), following a previously published work [85]. Cytokine concentrations were reported in pg/mL. The detection limits for each cytokine were determined from standard curves: IL-6 (4.7 pg/mL), IL-8 (31.2 pg/mL), TNF (7.8 pg/mL), and MIF (7.8 pg/mL).

### 5.6. Invasion and Attachment Assay

To investigate whether SVMPs act directly on parasites or host cells, impairing early steps of *T. gondii* infection, an invasion and attachment assay was performed as previously described [26], with minor modifications. HeLa cells were seeded at a density of 1.0 × 10^5^ in 24-well microplates containing 13 mm coverslips. In the first set of experiments, to investigate the possible protective effect of toxins on host cells, HeLa cells were pretreated for 30 min or 24 h at 37 °C and 5% CO_2_ with the culture medium (control group), Jar, or Jar-C (both 40 μg/mL). Next, the treatments were removed and parasites (MOI of 3:1) were allowed to interact with cells for 3 h at 37 °C and 5% CO_2_.

In the second set of experiments, we assessed the possible direct action of SVMPs on parasites. *T. gondii* tachyzoites (3:1) were added to microtubes and treated for 30 min at 37 °C and 5% CO_2_ with the culture medium (control group), Jar, or Jar-C (both 40 μg/mL). After that, parasites were centrifuged, resuspended in a treatment-free medium, and placed in 96-well microplates containing adhered HeLa cells (1.0 × 10^5^ cells) for 3 h at 37 °C and 5% CO_2_.

At the end of both experimental procedures mentioned above, cells were carefully rinsed with 1× PBS (Thermo Fisher Scientific, IL, USA) to remove the excess of non-invaded parasites and then fixed with 4% paraformaldehyde (PFA) (Fluka Chemika, Buchs, St. Gallen, Switzerland) for 12 min at room temperature. For labeling, fixed cells were submitted to rabbit polyclonal primary anti-*T. gondii* antibody (Abcam #20530; Waltham, MA, USA) [diluted 1:500 in PGN (PBS containing 0.25% gelatin)] for 1 h, followed by Alexa Fluor 594-conjugated anti-rabbit IgG (Invitrogen, USA #A11012; Waltham, MA, USA), also diluted 1:500 in PGN. Similarly, cells were incubated with rabbit polyclonal primary anti-*T. gondii* antibody (diluted 1:500 in PGN-0.01% saponin-permeabilizing solution, Sigma) for 1 h, followed by incubation for 1 h with Alexa Fluor 488-conjugated anti-rabbit IgG (Invitrogen, USA #A11008; Waltham, MA, USA), and the cell nucleus marker DAPI, both diluted in PGN + saponin. Finally, we mounted coverslips on glass slides, and samples were analyzed by confocal fluorescence microscopy (Zeiss, LSM 510 Meta, Oberkochen, Baden-Württemberg, Germany) with an inverted microscope (Zeiss Axiovert 200 M). The number of intracellular (green^+^/red^−^) and adhered [red or red^+^/green^+^ (yellow)] parasites was scored on 20 randomly selected fields on each separately mounted coverslip. We normalized the data by the ratio (number of parasites/cell nucleus) of each experimental condition.

### 5.7. Azocaseinolytic Activity

Before assessing the maintenance of the antiparasitic action of the SVMPs after inhibiting with the protease inhibitor EDTA (Sigma), we further confirmed the enzymatic inhibition of EDTA by testing the azocaseinolytic activity of toxins upon the azocasein substrate, as previously published [86]. Briefly, BpMP-I and Jar (5 μg)—both with enzymatic activity—were preincubated with 10 μL of EDTA (1 mM) for 1 h at 37 °C. As control, we also used the crude venoms (5 μg) of *B. pauloensis* and *B. jararaca*. Afterwards, 45 μL of this solution was incubated at 37 °C and 5% CO_2_ with 100 μL of azocasein (1 mg/mL) (Sigma) in 0.05 M Tris-HCl and 0.15 M NaCl (LGC Biotecnologia, SP, Brazil) in 96-well microplates. After 30 min, the reaction was stopped by adding 45 μL of trichloroacetic acid 20% (*m*/*v*) in each sample, which was incubated at room temperature for an additional 30 min and then centrifuged at 3000× *g* for 20 min (Eppendorf 5430R, Hamburg, Barkhausenweg, Germany). The absorbance of the supernatant was measured at 366 nm with a multi-well scanning spectrophotometer (Multiskan GO, Thermo). One unit (U) of azocaseinolytic activity was defined as an increase of 0.01 absorbance units per minute.

### 5.8. Analysis of the Influence of the Catalytic Activity of SVMPs on Impairing T. gondii Growth

To gain insights into the role of the catalytic activity of SVMPs in triggering their anti-*T. gondii* effect, we preincubated or not 40 μg/mL of BpMP-I, Jar, and Jar-C for 1 h at 37 °C with EDTA (1 mM). In parallel, adhered HeLa cells (3 × 10^4^/96-well microplate) were infected for 3 h at 37 °C with *T. gondii* tachyzoites (MOI of 3:1) and then treated for 24 h at 37 °C and 5% CO_2_ with the toxins preincubated with EDTA. As controls, infected cells were incubated with culture medium only, EDTA (1 mM), or SVMPs (40 μg/mL without EDTA pretreatment). *T. gondii* intracellular proliferation was carried out as described above (see item 5.4).

### 5.9. Molecular Modeling and Multiple Sequence Alignment

We performed a bioinformatics analysis to investigate the structure–function relationship of Jar and Jar-C and gain insights into the involvement of the protein domains in the control of *T. gondii* infection. The tertiary structures of Jar and Jar-C were built by homology modeling using SWISS-MODEL [87]. Briefly, the sequences of Jar (UniProt/SwissProt [88]: P30431) and Jar-C [30] were modeled using, as a template, the structure of Bothropasin [89] (PDB [90]: 3DSL) from *B. jararaca*. The protein models were refined using MolProbity [91], and QMEANDisCo [92] and ERRAT [93] were used to assess the stereochemical quality (Supplementary File S2) in SAVES v.6.0 (https://saves.mbi.ucla.edu/) (accessed on 27 February 2024). Subsequently, the three-dimensional structures of Jar and Jar-C were analyzed in PyMOL v. 2.5 (Schrödinger, Inc., New York, NY, USA), and the electrostatic surface was determined using the plugin APBS Electrostatics [94]. In addition, to verify the similarities between the sequences of Jar, Jar-C, and Bothropasin, a Multiple Sequence Alignment (MSA) was performed using Clustal Omega v. 1.4.2 [95] and visualized using the package pyMSAviz [96].

### 5.10. Molecular Dynamics

We performed molecular dynamics simulations to further explore the structures of Jar and Jar-C and the effect of EDTA in their structures. The 3D structure of EDTA was obtained from PubChem [97] under accession code 6049. The protonation states of EDTA at a pH of 7.4 were corrected using Avogadro v. 1.2.0 [98], and the protein protonation states were corrected with AMBER in APBS [99]. MD simulations were performed in GROMACS v. 2022 [100]. The parametrization of EDTA was performed using ACPYPE [101], and the proteins were parameterized using AMBER99SB [102]. The systems were simulated in a cubic box with 1.5 nm of limits in any dimension and filled with TIP3P water molecules [103]. The systems were neutralized by applying Na^+^ and Cl^−^ counterions. Then, a two-step energy minimization was performed using steepest descent and conjugate gradient algorithms. Next, atomic velocities were assigned using a Maxwell–Boltzmann distribution, and the systems were gradually heated to 300 K with a velocity-rescaling thermostat (V-rescale). Subsequently, an equilibration of pressure was conducted using the Parrinello–Rahman barostat to 1 atm. Afterwards, four MD productions were performed in three independent simulations each of 200 ns: (i) Jar; (ii) Jar-C; (ii) Jar + EDTA; and (iv) Jar-C + EDTA. The Root Mean Square Fluctuation (RMSF) was calculated fitting the C-alpha atoms, while the Root Mean Square Deviation (RMSD) and the radius of gyration were calculated fitting the backbone. The hydrogen bonds were calculated considering a maximum distance of 3.5 Å between donor and acceptor, and a maximum angle of 30° between donor, donor–hydrogen, and acceptor. The trajectories were analyzed and visualized using VMD (Visual Molecular Dynamics) v. 1.9.3 [104], and the images were generated in PyMOL v.2.5 (Schrödinger, Inc., NY, USA).

### 5.11. Statistical Analysis

Data were expressed as mean ± standard error of the mean (±SEM) using GraphPad Prism version 8.01. Significant differences were compared to controls by using one-way ANOVA. The parametric data were analyzed by Bonferroni’s multiple comparisons post-test and the Kruskal–Wallis test and Tukey’s multiple comparison post-test for non-parametric. Values of *p* < 0.05 were considered statistically significant.

## Data Availability

The original contributions presented in this study are included in this article and Supplementary Material. Further inquiries can be directed to the corresponding authors.

## Supplementary Materials

The following supporting information can be downloaded at: https://www.mdpi.com/article/10.3390/toxins17020095/s1, Supplementary File S1. SDS-PAGE. Supplementary File S2. Quality assessment of Jar and Jar-C structures. Supplementary File S3. Multiple Sequence Alignment (MSA). Supplementary File S4. Molecular dynamics of Jar. Supplementary File S5. Molecular dynamics of Jar with EDTA. Supplementary File S6. Molecular dynamics of Jar-C. Supplementary File S7. Molecular dynamics of Jar-C with EDTA.
