# Supplementary material for: Insights into the Role of Proteolytic and Adhesive Domains of Snake Venom Metalloproteinases from Bothrops spp. in the Control of Toxoplasma gondii Infection
Source: Toxins (Basel). 2025 Feb 18;17(2):95. doi: 10.3390/toxins17020095 (PMC11861417; doi:10.3390/toxins17020095)

Supplementary File S1. SDS-PAGE.

The analysis of protein homogeneity was evaluated by SDS-PAGE electrophoresis under denaturing conditions (SDS), with minor modifications. BpMP-I is a P-I SVMP of 20 kDa isolated from *B. pauloensis* snake venom. Jar is a P-III SVMP with an estimated 52 kDa and Jar-C is a disintegrin-like protein of 28 kDa, both isolated from *B. jararaca* snake venom.

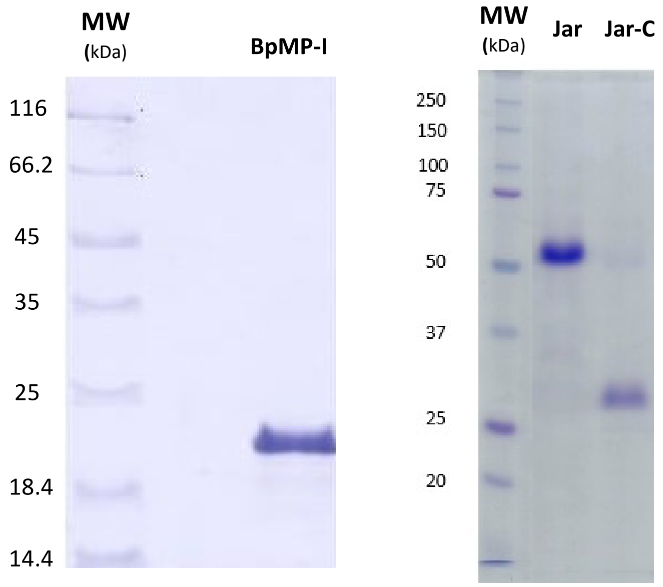

Supplement: Supplementary file 1 [file toxins-17-00095-s001.zip › toxins-3400005-supplementary/toxins_3400005_supplementary_fileS1_final.pdf]
