# Supplementary material for: Insights into the Role of Proteolytic and Adhesive Domains of Snake Venom Metalloproteinases from Bothrops spp. in the Control of Toxoplasma gondii Infection
Source: Toxins (Basel). 2025 Feb 18;17(2):95. doi: 10.3390/toxins17020095 (PMC11861417; doi:10.3390/toxins17020095)
Supplement: Supplementary file 1 [file toxins-17-00095-s001.zip › toxins-3400005-supplementary/toxins_3400005_supplementary_fileS2_final.pdf]

Supplementary File S2. Stereochemical quality of Jar and Jar-C models.

The predicted models of Jar and Jar-C showed a good stereochemical quality. QMEANDisCo validated the models with scores of 0.80 and 0.74 for Jar (**Figure S1**) and Jar-C (**Figure S2**), respectively. ERRAT pointed out a general quality factor of 93.81 and 87.07 for Jar (**Figure S3**) and Jar-C (**Figure S4**), respectively. MolProbity exhibited a good distribution of the atoms and torsional angles in the Ramachandran diagrams with 99.0% and 98.6% of the residues in allowed regions of Jar (**Figure S5**) and Jar-C (**Figure S6**), respectively

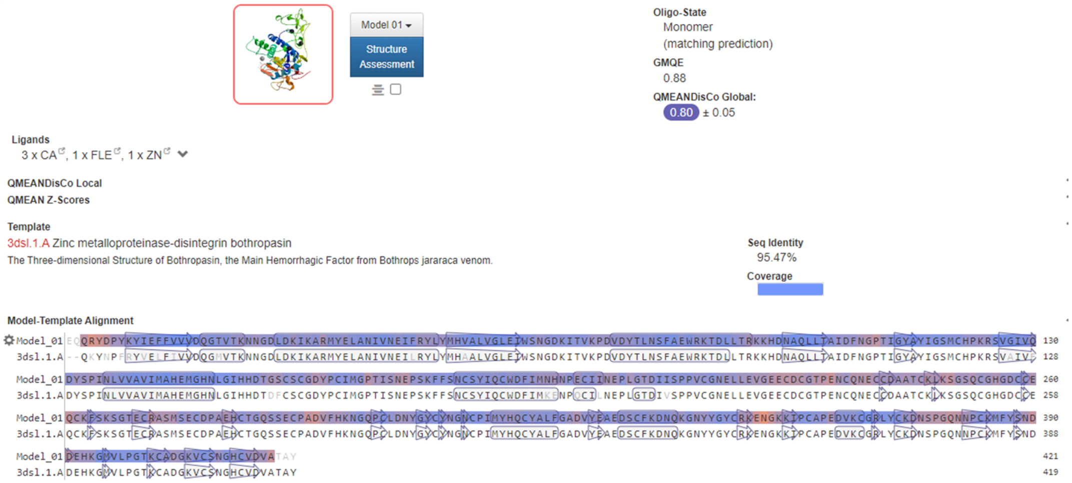

Figure S1. QMEANDisCo quality of Jar.

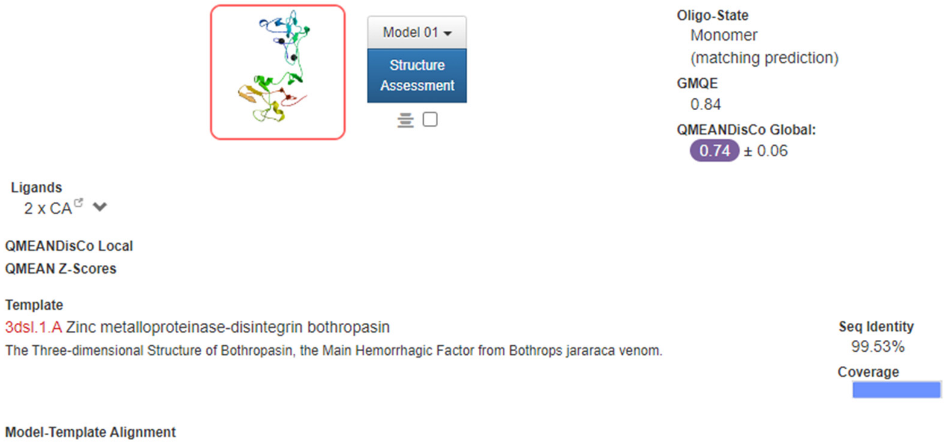

Figure S2. QMEANDisCo quality of Jar-C.

Program: ERRAT2
File: jararhaginFH.pdb
Chain#:A
Overall quality factor**: 93.812

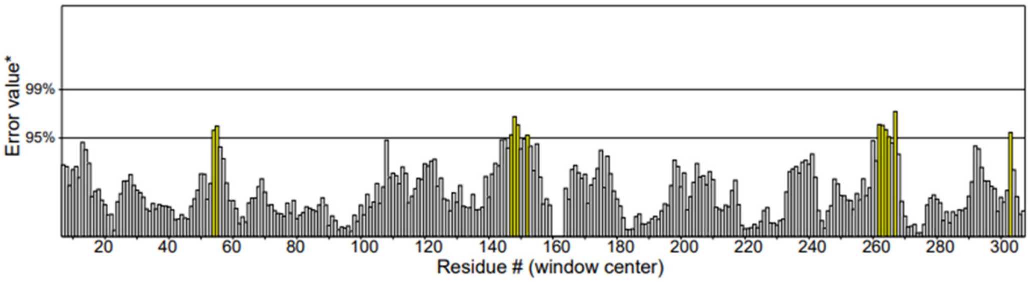

*On the error axis, two lines are drawn to indicate the confidence with which it is possible to reject regions that exceed that error value.

**Expressed as the percentage of the protein for which the calculated error value falls below the 95% rejection limit. Good high resolution structures generally produce values around 95% or higher. For lower resolutions (2.5 to 3A) the average overall quality factor is around 91%.

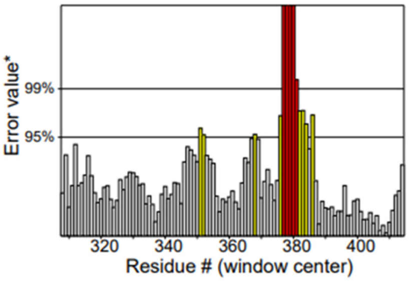

*On the error axis, two lines are drawn to indicate the confidence with which it is possible to reject regions that exceed that error value.

**Expressed as the percentage of the protein for which the calculated error value falls below the 95% rejection limit. Good high resolution structures generally produce values around 95% or higher. For lower resolutions (2.5 to 3A) the average overall quality factor is around 91%.

Figure S3. ERRAT quality of Jar.

Program: ERRAT2
File: jararhagin-CFH.pdb
Chain#:A
Overall quality factor**: 87.065

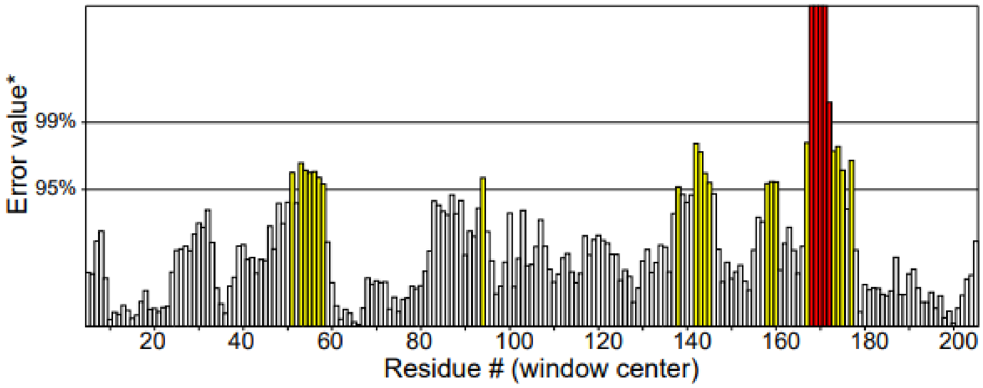

*On the error axis, two lines are drawn to indicate the confidence with which it is possible to reject regions that exceed that error value.

**Expressed as the percentage of the protein for which the calculated error value falls below the 95% rejection limit. Good high resolution structures generally produce values around 95% or higher. For lower resolutions (2.5 to 3A) the average overall quality factor is around 91%.

Figure S4. ERRAT quality of Jar-C.

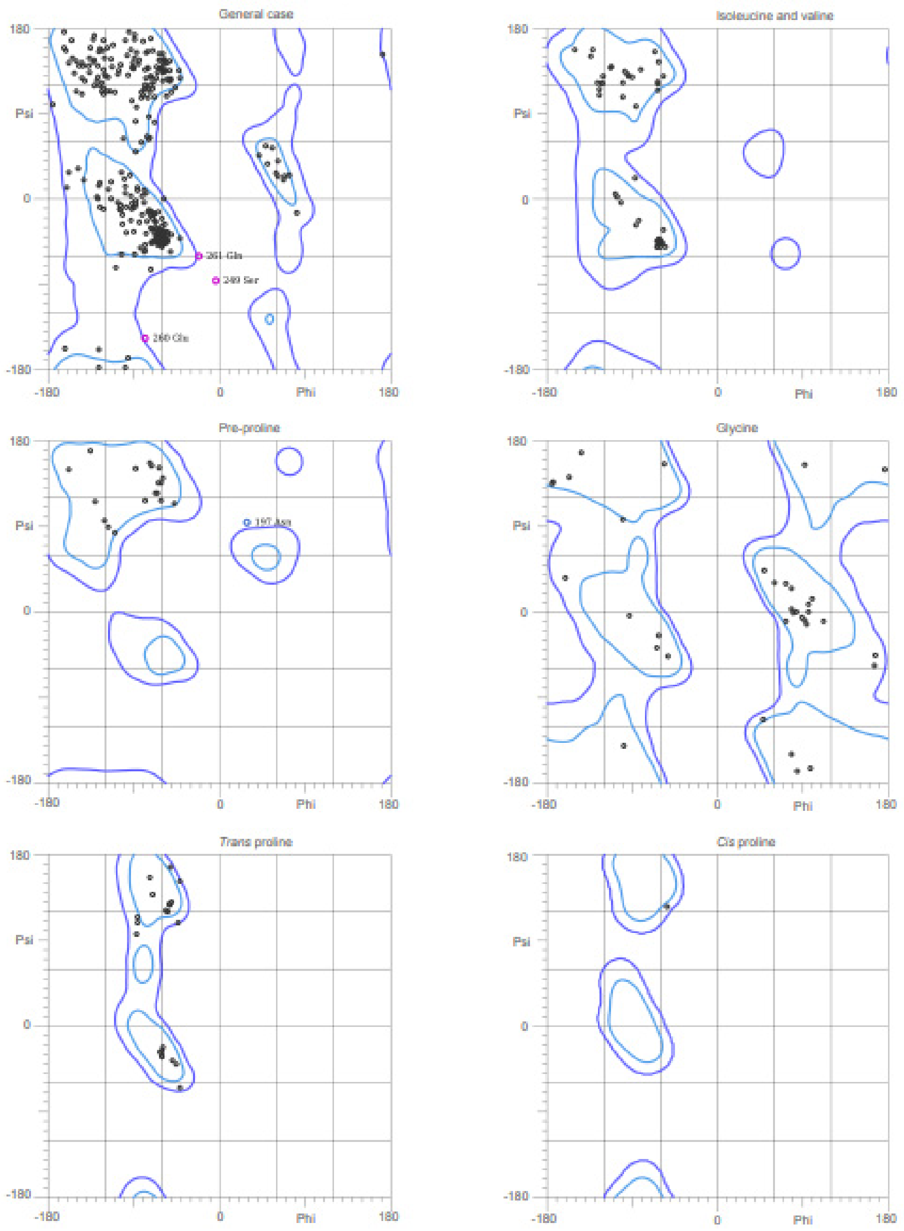

90.8% (376/414) of all residues were in favored (98%) regions.
99.0% (410/414) of all residues were in allowed (>99.8%) regions.

Figure S5. MolProbity quality of Jar.

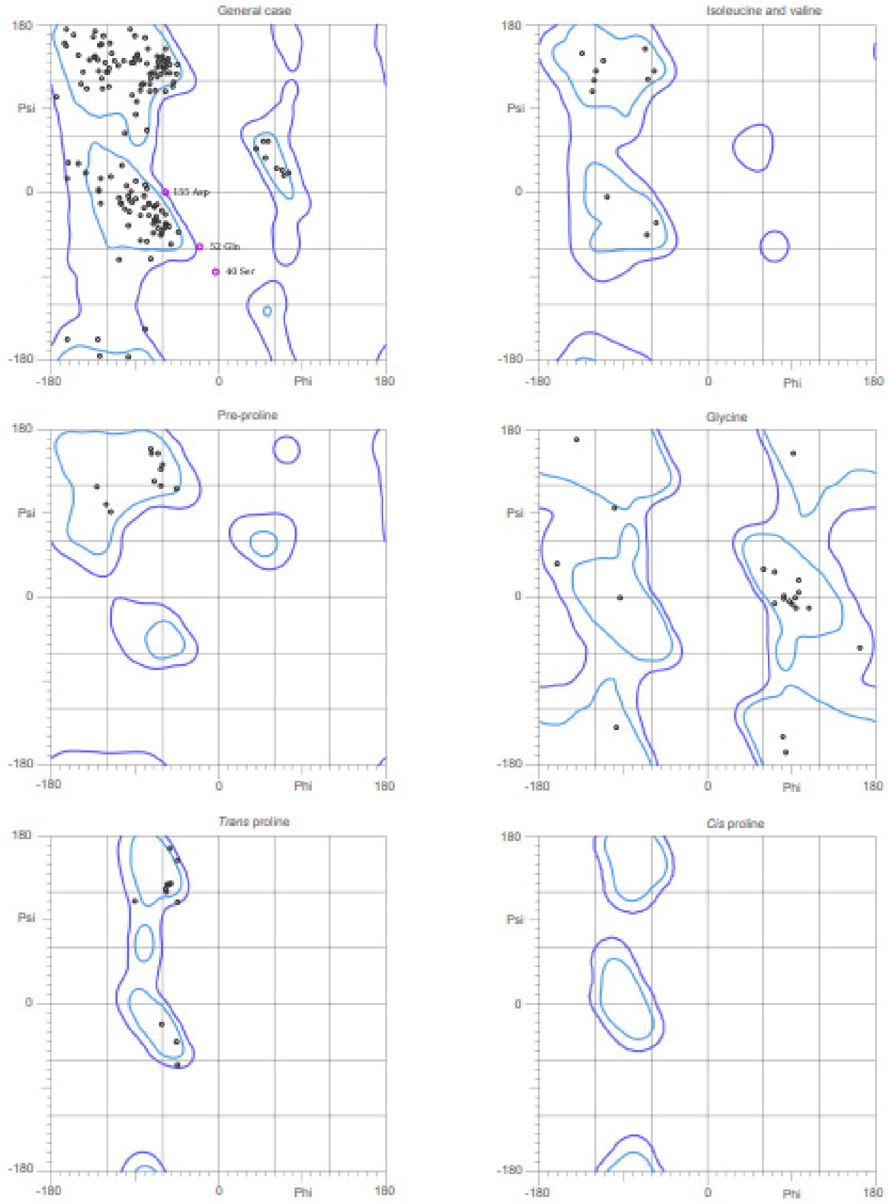

87.9% (182/207) of all residues were in favored (98%) regions.
98.6% (204/207) of all residues were in allowed (>99.8%) regions.

Figure S6. MolProbity quality model of Jar-C.
