# Supplementary material for: Insights into the Role of Proteolytic and Adhesive Domains of Snake Venom Metalloproteinases from Bothrops spp. in the Control of Toxoplasma gondii Infection
Source: Toxins (Basel). 2025 Feb 18;17(2):95. doi: 10.3390/toxins17020095 (PMC11861417; doi:10.3390/toxins17020095)

Supplementary File S3. Multiple Sequence Alignment (MSA).

The primary structure of Bothropasin, Jar and Jar-C aligned using Clustal Omega v. 1.4.2. The metalloproteinase (M), disintegrin-like (D) and cysteine-rich (C) domains are colored in pink, blue and orange, respectively. The mutations in the sequence of Jar and Jar-C in comparison with Bothropasin sequence are labeled by asterisks (▾). The Zn^{2+}-binding site (His^{145}-Glu^{146}-*X*-*X*-His^{149}-*X*-*X*-Glu^{152}-*X*-*X*-His^{155}) is colored in gray. The residues are colored according to Clustal Omega scheme: blue (hydrophobic – A, I, L, M, F, W, and C), red (positively charged – K and R), magenta (negatively charged – E and D), green (polar – N, Q, S, and T), pink (cysteine – C), Orange (glycines – G), yellow (prolines – P), cyan (aromatic – H and Y).

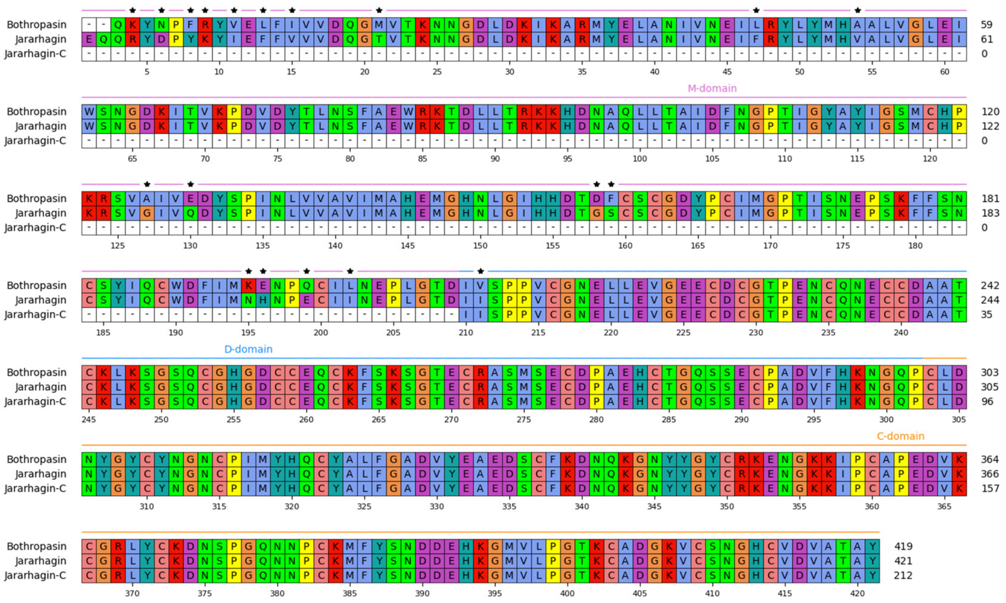

Supplement: Supplementary file 1 [file toxins-17-00095-s001.zip › toxins-3400005-supplementary/toxins_3400005_supplementary_fileS3_final.pdf]
